# Selective contralesional constructional hemi‐apraxia after unilateral brain damage: Which relationship with unilateral spatial neglect?

**DOI:** 10.1111/jnp.70006

**Published:** 2025-07-14

**Authors:** Francesco Panico, Angela Arini, Claudio Crisci, Luigi Trojano

**Affiliations:** ^1^ University of Campania “Luigi Vanvitelli” Caserta Italy; ^2^ Clinic Center Rehabilitation Institute Naples Italy

**Keywords:** constructional apraxia, drawing disorders, hyperschematia, unilateral brain damage, unilateral spatial neglect

## Abstract

We describe a peculiar contralesional drawing disorder in three patients affected by focal brain lesions, who did not show signs of unilateral neglect at standard clinical assessment, including the star cancellation test. This picture, that could be termed selective constructional hemi‐apraxia (CHA), could follow both right and left‐hemisphere lesions and is observed in complex constructional tasks. Future studies are warranted to explore the clinical and functional implications of CHA and its boundaries with unilateral neglect and constructional apraxia.

## INTRODUCTION

Acquired brain lesions, particularly to the right hemisphere, can determine visuo‐spatial deficits, including unilateral spatial neglect (USN) and constructional apraxia (CA). USN has been defined as a failure to report, respond, or orient towards stimuli contralateral to the brain lesion (Heilman & Valenstein, 1979), which can be observed in common cancellation and drawing tasks. Any impairment in drawing is currently encompassed within the operational definition of CA (Gainotti & Trojano, 2018), although the term was proposed by Kleist (1934) to identify a specific disorder of drawing and assembling in which the spatial form of the product is altered. Neural networks distributed across both hemispheres are involved in drawing in healthy individuals (Raimo et al., 2021), so that disparate brain lesions can induce CA, with possibly specific error patterns (Chen et al., 2016). One instance of such patterns is hyperschematia (Rode et al., 2014), described as a systematic expansion of drawings or of 3D assembling tasks, frequently contralateral to lesions in the right hemisphere.

Here we describe a selective visuo‐spatial alteration in the contralateral side of space following unilateral brain damage, in three patients affected by focal vascular brain lesions without evident asymmetries on standard USN assessment, including star cancellation. This condition could be termed as selective ‘constructional hemi‐apraxia’ (CHA) and has been only anecdotally reported in cognitive rehabilitation settings (Grossi, Lepore, & Trojano, 1999).

## METHOD

### Case description

We collected data from three patients who, at the end of their rehabilitation training, showed unusual asymmetry in copying complex figures. Informed consent to collect findings from their clinical evaluation was obtained by all three patients.

#### Case 1

**FIGURE 1:**
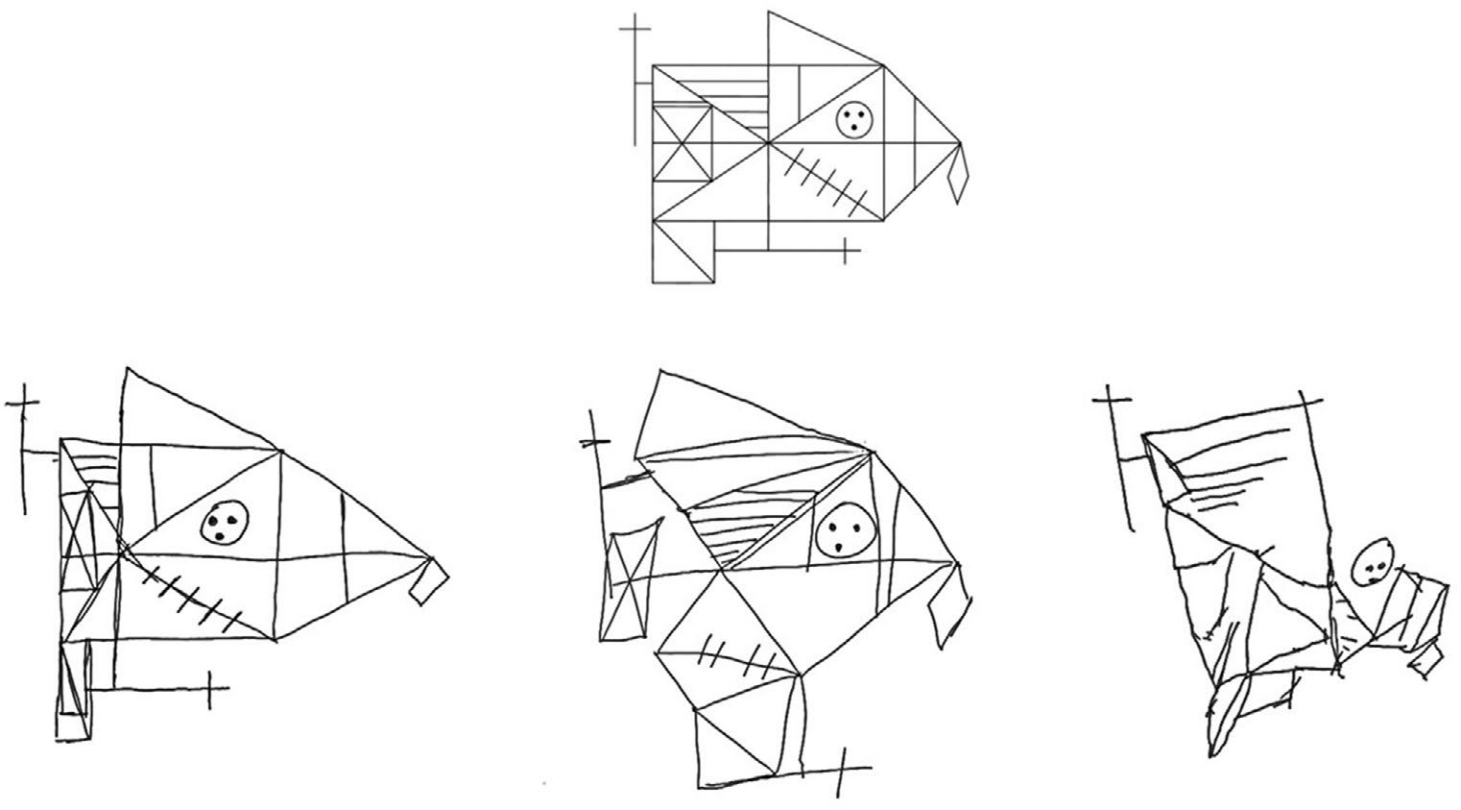
Copy of Rey–Osterreith figure by the three patients (from left to right: A.P., M.C.G and A.M.S.) describing constructional errors in the left (A.P. and M.C.G) or right contralesional space (A.M.S.) during a complex constructional task. Drawings were made using the right dominant hand.

**FIGURE 2:**
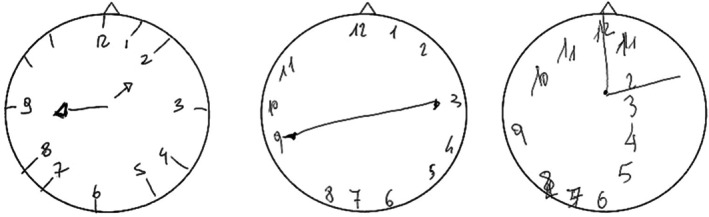
Clock drawing test by the three patients (from left to right: A.P., M.C.G and A.M.S.) describing omissions and errors in spatial allocation in the left (A.P. and M.C.G) or right contralesional space (A.M.S.) during a representational/constructional task. Drawings were made using the right dominant hand.

A.P. is a 37‐year‐old right‐handed male import–export entrepreneur. In July 2015, he suffered a haemorrhagic stroke involving the right posterior temporo‐parietal region resulting in moderate left USN, and attention and episodic memory impairments (only narrative descriptions are available). Seven years later A.P. came to our observation after having completed several cognitive rehabilitation trainings. At that time (October 2022), A.P. showed residual long‐term anterograde episodic memory impairments, and few erratic omissions on cancellation tasks without a clear‐cut left/right asymmetry; no asymmetry was observed in the exploration of own body (Table S1) or in completing Raven's Progressive Matrices. A.P. showed residual constructional disorders but no upper limb apraxia (Table S2). Copying simple stimuli was preserved (Figure S1), while copying a complex figure was characterised by spatial distortions, including spatial compression of the left part of the figure, positioning errors and one omission of a line in the left side (Figure 1). Clock drawing task was performed by an effective strategy in number spatial allocation (first arranging the 12–6 and 3–9 axes); here the patient correctly identified all number positions (the inner marks) but omitted two numbers on the upper‐left section of the dial (Figure 2).

#### Case 2

M.C.G. is a 51‐year‐old right‐handed male lawyer. In August 2024, he suddenly developed left hemiplegia due to a right fronto‐temporo‐parietal haemorrhagic stroke. At that time M.C.G. showed moderate left USN and impairments in executive functions, and underwent a rehabilitation programme focused on visual scanning and cognitive training. At the end of the treatment (November 2024), M.C.G. showed no upper limb apraxia, no omissions on cancellation and reading tasks or on own body exploration, whereas he still performed poorly in a cognitive estimation task (Tables S1 and S2). In copying simple stimuli, he showed no constructional deficits (Figure S1), which instead became evident in copying the left contralesional side of a complex figure, wherein marked spatial distortions were present in contrast with a better spatially organised right side (Figure 1); the elements in the left side of the figure (but the left side of the large rectangle) were reproduced but mainly positioning errors were observed with the left square item and the left half of the main rectangle respectively shifted and compressed to the bottom. Clock drawing was characterised by an impairment in number spatial allocation in the left half of the dial, whereas number positioning in the right half was relatively spared (Figure 2).

#### Case 3

A.M.S. is a 58‐year‐old right‐handed female cashier. In January 2024, the patient developed a brain haemorrhage due to the rupture of an arteriovenous malformation in the left parieto‐occipital region, which was treated surgically. Upon hospital discharge (March 2024) A.M.S. showed drawing disorders, executive dysfunctions and recall difficulties in episodic memory tasks. In the cancellation and drawing tasks no omissions were observed, but the patient placed her drawing productions close to the left margin of the response sheet. At retest (May 2024) the patient showed a modest improvement in her overall cognitive profile and no upper limb apraxia (Tables S1 and S2); her performance on Raven's Progressive Matrices was still defective, but without clear asymmetries in responses. A.M.S. did not show difficulties in copying simple figures (Figure S1), apart from a residual tendency to place her drawings towards the left margin of the sheet, but in copying a complex stimulus she produced several spatial distortions in the right contralesional space, involving positioning errors, omissions and spatial compression (Figure 1). In clock drawing A.M.S. exhibited an altered allocation of the numbers within the dial, prominently in the right contralesional space (Figure 2).

In summary, all three patients showed a clearly asymmetric visuo‐spatial impairment in the contralesional side, characterised by spatial distortions in complex constructional tasks, without marked asymmetries in standard USN evaluation, including star cancellation.

### Rehabilitative training

The patients described above (as well as the small group of control patients, see below) received a cognitive treatment for USN consisting of (i) visual–spatial scanning, (ii) sentence reading, (iii) picture description and (iv) copying matrices and complex figures according to the principles provided by Pizzamiglio et al. (1992). M.C.G. and A.M.S. underwent daily 40‐min sessions (Monday to Friday) for 8 weeks; rehabilitative treatment for A.M.S. posed particular emphasis on graphic tasks. Patient A.P. had completed three rehabilitative cycles, with a similar approach (only narrative description available), before he arrived at our observation and CHA was noticed.

### Data analysis

To quantify asymmetries in constructional errors, we developed a specific scoring procedure for the Rey–Osterreith figure (see Data S1)—considering positioning errors, spatial compression of the items and partial reproduction of the elements—to obtain a laterality index (LI) based on Veronelli et al. (2020) and Rode et al. (2014) [LI: (left‐sided errors minus right‐sided errors/left‐sided errors plus right‐sided errors) × 100], with negative values meaning more errors in the affected side (left for A.P. and M.C.G, right for A.M.S., whose LI score was reversed). The index obtained by each of the three patients was compared by means of the Crawford case–control method (SINGLIMS_ES programme; Crawford et al., 2010) with those obtained by a small group of patients (*n* = 5; 2 female, age range 40–60), consecutively admitted to our rehabilitative unit and who had recovered from USN following a rehabilitative treatment (neuropsychological and clinical data of control patients are provided in Table S3).

## RESULTS

**FIGURE 3:**
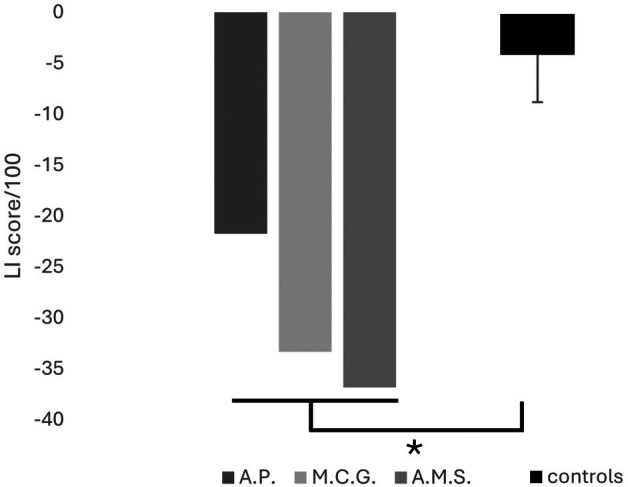
LI scores for constructional errors exhibited by the three patients with CHA (A.P., M.C.G. and A.M.S.) and the control group of patients without CHA (*n* = 5; mean ± SD) in the copy of the Rey–Osterreith figure. Negative values indicate higher asymmetry in constructional errors. The Crawford case‐control tests revealed significant differences (* = *p* < .05) for all three patients: A.P.: t = −3.005, *p* = .019, effect size = −3.29, confidence interval = −5.64 to −.94; M.C.G: t = −5.02, *p* = .004, effect size = −5.5, confidence interval = −9.27 to −1.79; A.M.S.: t = −5.64, *p* = .002, effect size = −6.17, confidence interval = −10.38 to −2.03.

LI scores of each of the three patients with CHA (A.P.: −21.74, M.C.G.: −33.33, A.M.S.: −36.84) revealed a significantly higher number of constructional errors in the affected side with respect to the control group (*M* = −4.49, SE = 5.24; Figure 3). Estimated percentage of control population falling below each patient's score (Crawford et al., 2010) was 1.98%, .37% and .24% respectively.

## DISCUSSION

Although contralesional omissions and spatial distortions in drawing are often observed in patients with USN, the present report outlines a picture of selective CHA in the lack of clear asymmetries in clinical neglect assessment, including star cancellation tasks. Selective CHA seems to be characterised by three main features. First, it may follow both right (patients A.P. and M.C.G) and left‐hemisphere lesions (patient A.M.S.) involving the parietal, frontal or temporal regions, in line with the current understanding of drawing disorders as the by‐product of dysfunctions in complex brain networks (Gainotti & Trojano, 2018).

Second, selective CHA seems to be associated with the constructional load. Indeed, the present patients performed well (and without omissions) when copying simple figures, whereas they made a significant number of asymmetric errors in more demanding constructional tasks. This may suggest that CHA reveals in constructional tasks in which the graphic productions cannot solely rely on the activation of previously stored constructional schemata (i.e. a sort of ‘constructional lexicon’; Grossi, Lepore, & Trojano, 1999), but are build piece‐by‐piece and require the mental representation/processing of model's spatial relationships (Trojano et al., 1993). This is also consistent with the asymmetric performance of patients M.C.G and A.M.S. in the clock drawing task, which showed defective spatial positioning of the items in the contralesional half of the dial.

Third, as CHA appears on the contralesional side, it could be considered a USN‐related phenomenon, resembling other distortions of size or spatial relationships on contralesional graphic productions (e.g. Grossi, Lepore, Esposito, et al., 1999). The three patients described here developed some degree of USN but, at the time of CHA observation, clinically evident asymmetries on standard USN assessment, including star cancellation tasks, had recovered. However, not all patients who recover from USN exhibit CHA, as evidenced by the comparisons between our CHA patients and the control group. Therefore, selective CHA may indicate the presence of visuo‐spatial alterations confined to the affected space when no omissions are obvious in star cancellation or simple copying tasks. It is possible that our patients had recovered a relatively symmetrical exploration of the space but still presented some distortion in processing/representing its contralesional side. Future studies could explore the relationship between CHA and USN, as CHA may be useful in the appreciation of residual functional impairment after USN recovery (Della Sala et al., 2018). In addition, although we described CHA after USN recovery, it is entirely possible that CHA can go unnoticed in the acute stage in patients without evident USN. Conversely, it could be difficult to isolate selective contralesional errors during the acute USN stage due to the prevalence of omissions errors in this phase or severe CA. For instance, in the pre‐treatment assessment of Rey–Osterreith figure copying, our patient M.C.G. produced a very poor figure, with a few perseverative elements and clear left‐sided omissions, not viable for a reliable analysis of constructional asymmetries, whereas patient A.M.S. produced a severely distorted figure with many errors in positioning individual elements and spatial transpositions, so that the resulting drawing could not be easily scored for asymmetries. The scoring procedure we developed to quantify asymmetries in the Rey–Osterreith figure could reveal useful for systematic assessment of graphic productions in brain damaged and healthy individuals.

It is worth mentioning that another asymmetric constructional disorder, hyperschematia, has been considered as independent from USN (Rode et al., 2014) and described in the contralesional and, later, in the ipsilesional side (Veronelli et al., 2020). Further evidence should ascertain whether this also applies to CHA and whether there is any relationship with hyperschematia. Theoretically, the disproportionate enlargement of the left side of drawings in hyperschematia was ascribed to a horizontal anisometry of the medium for the representation of spatial relationships (Rode et al., 2014). CHA shares with hyperschematia a lateralised alteration of the spatial relationships, but here the impairment seems to consist in a specific inability to manage contralesional spatial coordinates and produce ‘spatially formed outputs’ (in Kleist's terms, Kleist, 1934; Trojano, 2020). Further cues for comprehending the nature of these asymmetric drawing disorders could be provided by studies ascertaining whether they can be modulated by specific interventions for CA (Grossi, Lepore, & Trojano, 1999) or USN (Panico et al., 2022).

It is important to acknowledge some limitations of this brief clinical report. First, although the star cancellation test combined with copying tests constitutes a clinically meaningful method to assess USN (Halligan et al., 1989, 1990; Klinke et al., 2018), a more detailed assessment would contribute to ascertaining subtle residual signs of USN in the experimental studies necessary for establishing the boundaries between CHA, USN and CA. Second, it is important to remark that constructional performance as assessed by two‐ and three‐dimensional tasks requires a plethora of functions involving visuo‐perceptual, mental representation, motor ideation/implementation and executive processes differently affected by specific lesional profiles (Gainotti & Trojano, 2018). Although none of the patients described here presented manifest ideomotor or ideational apraxia, we cannot exclude that impairments in motor ideation/implementation might partially modulate the observation of CHA. Similarly, future studies could explicitly address the contribution of specific visuo‐spatial components of the working memory system (Baddeley, 2003), like the visuospatial sketchpad, which has been demonstrated to affect constructional performance in copying and spontaneous drawing (Papagno, 2002). The clinical nature of the present brief report did not allow full assessment of patients' features, which will be essential to untangle CHA rigorously.

## AUTHOR CONTRIBUTIONS

**Francesco Panico:** Conceptualization; investigation; writing – original draft; methodology; validation; visualization; writing – review and editing; software; formal analysis; project administration; data curation. **Angela Arini:** Conceptualization; investigation; writing – review and editing. **Claudio Crisci:** Conceptualization; investigation; writing – review and editing. **Luigi Trojano:** Conceptualization; investigation; funding acquisition; methodology; writing – review and editing; supervision.

## CONFLICT OF INTEREST STATEMENT

The authors report there are no competing interests to declare.

## Supporting information


Table S1.

## Data Availability

The data that support the findings of this study are available from the corresponding author upon reasonable request.
