# Supplementary material for: Selective contralesional constructional hemi‐apraxia after unilateral brain damage: Which relationship with unilateral spatial neglect?
Source: J Neuropsychol. 2025 Jul 14;20(1):284–90. doi: 10.1111/jnp.70006 (PMC12976822; doi:10.1111/jnp.70006)
Supplement: Supplementary file 1 — Table S1. [file JNP-20-284-s001.docx]

**Supplementary Files**

**Selective Contralesional Constructional Hemi-Apraxia after Unilateral Brain Damage: which relationship with unilateral spatial neglect?**

**Scoring procedure for asymmetry at the Rey-Osterreith Complex Figure**

To calculate asymmetry for constructional errors in the copy of the Rey-Osterreith Figure, we identified the midline vertical axis of entire Figure, roughly overlapping with item 10 in the original scoring system (Caffarra et al., 2002) and not with the midline of the main rectangle, to capture best right and left extension of the Figure, independently from the drawing strategy adopted by single individuals (most patient with brain-lesions do not adopt a ‘global’ drawing strategy; Trojano et al. 1993).


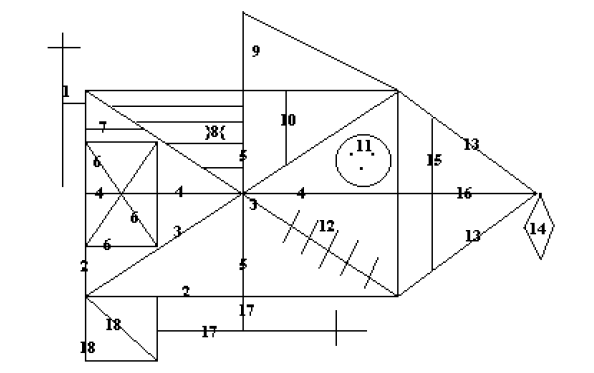


This allowed to classify the items into right- (n=6) and left- (n=6) located elements, and some central elements (n=5) whose right- and left- parts were scored separately (item 10 not considered in the scoring procedure). Each item was scored 0-2 points based on positioning and completeness of the element, as described in the classic scoring procedure (Caffarra et al., 2002). The maximum score for the right- and left- located items was 22 (total score 44).

The notation sheet reported below, describing the items allocation in the right- and left- positions and the achievable scores, was used to assess patients’ drawings:

| Left | | Centre | | | | Right | |
| --- | --- | --- | --- | --- | --- | --- | --- |
|  |  | Left part | | Right part | |  |  |
| Item | Score | Item | Score | Item | Score | Item | Score |
| 1 |  | 2a |  | 2b |  | 11 |  |
| 5 |  | 3a |  | 3b |  | 12 |  |
| 6 |  | 4a |  | 4b |  | 13 |  |
| 7 |  | 9a |  | 9b |  | 14 |  |
| 8 |  | 17a |  | 17b |  | 15 |  |
| 18 |  | **/10** | | **/10** | | 16 |  |
| **/12** | |  |  |  |  | **/12** | |
| **Total left** | | **/22** | | **Total right** | | **/22** | |

**Supplementary Table 1**. Assessment of Unilateral Spatial Neglect and performance in constructional tasks at baseline and at time of CHA observation

| **Neglect test** |  |  | | | | | | |
| --- | --- | --- | --- | --- | --- | --- | --- | --- |
|  |  | **Baseline** | | |  | **At time of CHA** | | |
|  |  | *Patient 1* | *Patient 2* | *Patient 3* |  | *Patient 1* | *Patient 2* | *Patient 3* |
| Star cancellation: right omissions |  | - | 3/27 | 0/27 |  | 2/27 | 0/27 | 0/27 |
| Star cancellation: left omissions |  | - | 25/27 | 0/27 |  | 2/27 | 0/27 | 0/27 |
| Sentence reading: omissions |  | - | 0/6 | 0/6 |  | 0/6 | 0/6 | 0/6 |
| Comb and razor task |  | - | mild asymmetry | no asymmetry |  | no asymmetry | no asymmetry | no asymmetry |
| Copying drawings |  | - | 3/14 | 8/14 |  | 10/14 | 10/14 | 8/14 |
| Rey’s figure: copy |  | - | 2/36 | 10/36 |  | 27/36 | 19.5/36 | 8/36 |
| Clock drawing test |  | - | 4/10 | 4/10 |  | 8/10 | 7/10 | 1/10 |

**Supplementary Table 2**. Neuropsychological assessment of the patients at time of CHA observation

| **Neuropsychological test** | *Patient 1* | | *Patient 2* | | *Patient 3* | |
| --- | --- | --- | --- | --- | --- | --- |
|  | Raw score | ES | Raw score | ES | Raw score | ES |
| Montreal Cognitive Assessment | 22/30 | 3 | 26/30 | 4 | 18/30 | 2 |
| Digit span | 6 | 4 | 6 | 4 | 6 | 4 |
| Verbal span | 4 | 1 | 4 | 1 | 4 | 2 |
| Babcock Story Recall | 6,3/16 | **0** | 10,7/16 | 2 | 6,7/16 | 1 |
| Rey’s 15 words: immediate recall | 34/75 | **0** | 52/75 | 4 | 35/75 | 2 |
| Rey’s 15 words: delayed recall | 4/15 | **0** | 13/15 | 4 | 3/15 | **0** |
| Rey’s figure: reproduction from memory | 7,5/36 | **0** | 4/36 | **0** | 3/36 | **0** |
| Raven's coloured progressive matrices | 31/36 | 3 | - | - | 17/36 | **0** |
| Cognitive estimation test: errors | 16/42 | N | 24/42 | **P** | - | - |
| Cognitive estimation test: bizzarries | 5/21 | **P** | 6/21 | **P** | - | - |
| Phonological fluency | 46 | 4 | 43 | 4 | 31 | 4 |
| Frontal Assessment Battery | 18/18 | 4 | 18/18 | 4 | 17/18 | 4 |
| Meaningful gesture imitation | 5/5 | # | 5/5 | # | 5/5 | # |
| Meaningless gesture imitation | 5/5 | # | 5/5 | # | 5/5 | # |
| Use of actual objects | 5/5 | # | 5/5 | # | 5/5 | # |

*Note:* ES indicates Equivalent Scores in the Italian scoring system; 0= means a pathological performance, while ES equal or above 1 indicates a performance falling in the normative range. For the tests not using the ES system, P indicates a pathological performance, and N indicates a normal performance at the test. Pathological scores highlighted in bold; # non standardised tasks.

**Supplementary Table 3**. Neuropsychological assessment and clinical data of control patients without CHA

| **Neuropsychological test** | *Patient 1* | | *Patient 2* | | *Patient 3* | | *Patient 4* | | *Patient 5* | |
| --- | --- | --- | --- | --- | --- | --- | --- | --- | --- | --- |
|  | Raw score | ES | Raw score | ES | Raw score | ES | Raw score | ES | Raw score | ES |
| Montreal Cognitive Assessment | 25/30 | 4 | 21/30 | 2 | 19/30 | 3 | 25/30 | 4 | 26/30 | 4 |
| Digit span | 6 | 4 | 6 | 4 | 5 | 4 | 4 | 2 | 6 | 4 |
| Verbal span | 6 | 4 | 4 | 1 | 4 | 2 | 6 | 4 | 5 | 4 |
| Babcock Story Recall | 12.2/16 | 2 | 15.6/16 | 4 | 9.5/16 | 2 | 11.5/16 | 2 | 11.7/16 | 4 |
| Rey’s 15 words: immediate recall | 45/75 | 4 | 27/75 | **0** | 35/75 | 2 | 36/75 | 2 | 56/75 | 4 |
| Rey’s 15 words: delayed recall | 9/15 | 4 | 4/15 | **0** | 7/15 | 3 | 4/15 | **0** | 10/15 | 4 |
| Copying drawings | 13/14 | 4 | 12/14 | 2 | 9/14 | 1 | 14/14 | 4 | 14/14 | 4 |
| Rey’s figure: copy | 35/36 | 4 | 28/36 | **0** | 30/36 | 2 | 29/36 | 1 | 33/36 | 4 |
| Rey’s figure: reproduction from memory | 12.5/36 | 3 | 5.5/36 | **0** | 7/36 | **0** | 6/36 | **0** | 13/36 | 2 |
| Clock drawing test | 8/10 | N | 7/10 | P | 7/10 | N | 10/10 | N | 9/10 | N |
| Raven's coloured progressive matrices | 27/36 | 3 | 28/36 | 3 | - | **-** | 29/36 | 3 | 27/36 | 2 |
| Phonological fluency | 51 | 4 | 23 | 1 | 24 | 3 | 29 | 4 | 41 | 4 |
| Frontal Assessment Battery | 15/18 | 2 | 16/18 | 1 | 13/18 | 1 | 14/18 | 1 | 13/18 | 4 |
| Star cancellation: right omissions | 1/27 | # | 0/27 | # | 2/27 | # | 0/27 | # | 0/27 | # |
| Star cancellation: left omissions | 2/27 | # | 0/27 | # | 1/27 | # | 0/27 | # | 0/27 | # |
| Sentence reading: omissions | 0/6 | # | 0/6 | # | 0/6 | # | 0/6 | # | 0/6 | # |
| Comb and razor task | no asymmetry | # | no asymmetry | # | no asymmetry | # | no asymmetry | # | no asymmetry | # |
| *Lesion site* | right posterior parietal | | right fronto-parietal | | right temporo-parietal | | right nucleo-capsular | | right temporo-parietal | |

*Note:* ES indicates Equivalent Scores in the Italian scoring system; 0= means a pathological performance, while ES equal or above 1 indicates a performance falling in the normative range. For the tests not using the ES system, P indicates a pathological performance, and N indicates a normal performance at the test. Pathological scores highlighted in bold. #: ES not available for neglect assessment tests.


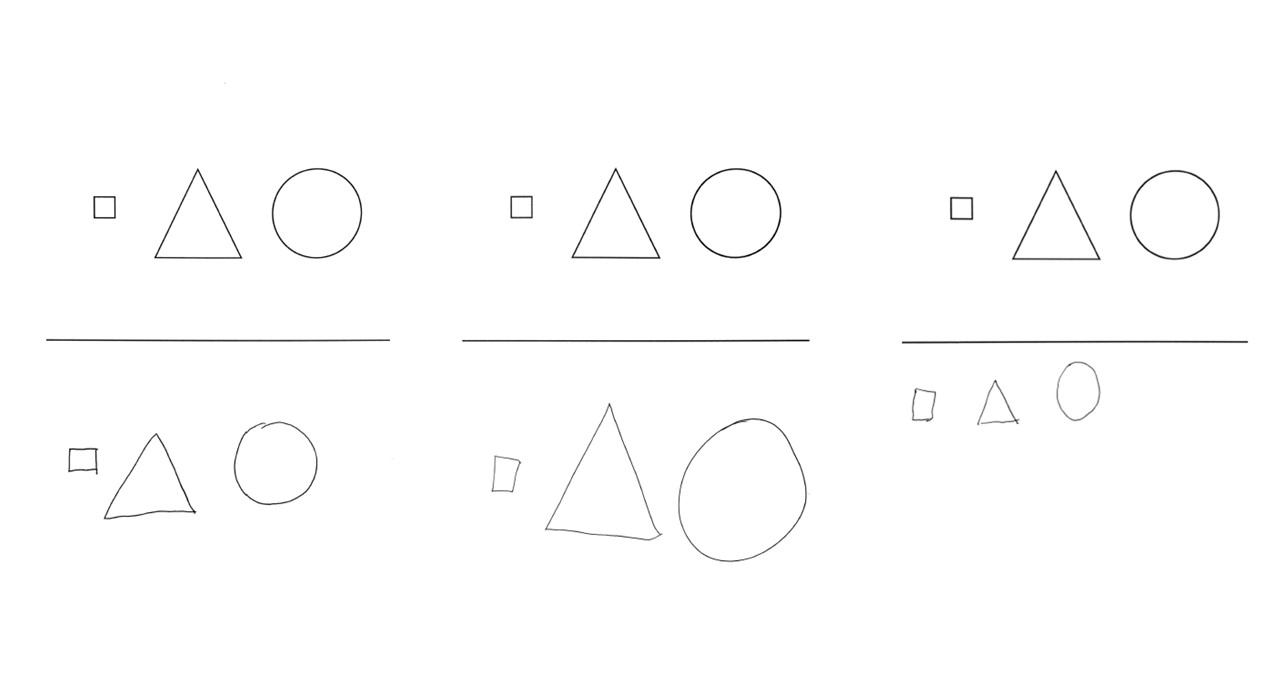


**Supplementary Figure 1**. Copy of simple figures by the three patients (from left to right: A.P., M.C.G, and A.M.S.) describing preserved performance in simple constructional tasks. Drawings were made using the right dominant hand.
